# Randomised Controlled Trial Of The Effect Of Tai Chi On Postural Balance Of People With Dementia

**DOI:** 10.2147/CIA.S228931

**Published:** 2019-11-19

**Authors:** Samuel R Nyman, Wendy Ingram, Jeanette Sanders, Peter W Thomas, Sarah Thomas, Michael Vassallo, James Raftery, Iram Bibi, Yolanda Barrado-Martín

**Affiliations:** 1 Department of Psychology and Ageing & Dementia Research Centre, Faculty of Science and Technology, Bournemouth University, Poole House, Talbot Campus, Poole, Dorset BH12 5BB, UK; 2 Peninsula Clinical Trials Unit, Peninsula Medical School, University of Plymouth, Plymouth, Devon PL4 8AA, UK; 3 Bournemouth University Clinical Research Unit, Faculty of Health and Social Sciences, Bournemouth University, Royal London House, Lansdowne Campus, Bournemouth, Dorset BH1 3LT, UK; 4 Centre of Postgraduate Medical Research and Education, Faculty of Health and Social Sciences, Bournemouth University, Royal London House, Lansdowne Campus, Bournemouth, Dorset BH1 3LT, UK; 5 Faculty of Medicine, University of Southampton, Highfield Campus, Southampton, SO17 1BJ, UK

**Keywords:** accidental falls, clinical trial, cognitive impairment, exercise, intervention

## Abstract

**Purpose:**

To investigate the effect of Tai Chi exercise on postural balance among people with dementia (PWD) and the feasibility of a definitive trial on falls prevention.

**Patients and methods:**

Dyads, comprising community-dwelling PWD and their informal carer (N=85), were randomised to usual care (n=43) or usual care plus weekly Tai Chi classes and home practice for 20 weeks (n=42). The primary outcome was the timed up and go test. All outcomes for PWD and their carers were assessed six months post-baseline, except for falls, which were collected prospectively over the six-month follow-up period.

**Results:**

For PWD, there was no significant difference at follow-up on the timed up and go test (mean difference [MD] = 0.82, 95% confidence interval [CI] = −2.17, 3.81). At follow-up, PWD in the Tai Chi group had significantly higher quality of life (MD = 0.051, 95% CI = 0.002, 0.100, standardised effect size [ES] = 0.51) and a significantly lower rate of falls (rate ratio = 0.35, 95% CI =0.15, 0.81), which was no longer significant when an outlier was removed. Carers in the Tai Chi group at follow-up were significantly worse on the timed up and go test (MD = 1.83, 95% CI = 0.12, 3.53, ES = 0.61). The remaining secondary outcomes were not significant. No serious adverse events were related to participation in Tai Chi.

**Conclusion:**

With refinement, this Tai Chi intervention has potential to reduce the incidence of falls and improve quality of life among community-dwelling PWD [Trial registration: NCT02864056].

## Video abstract

**Figure:**
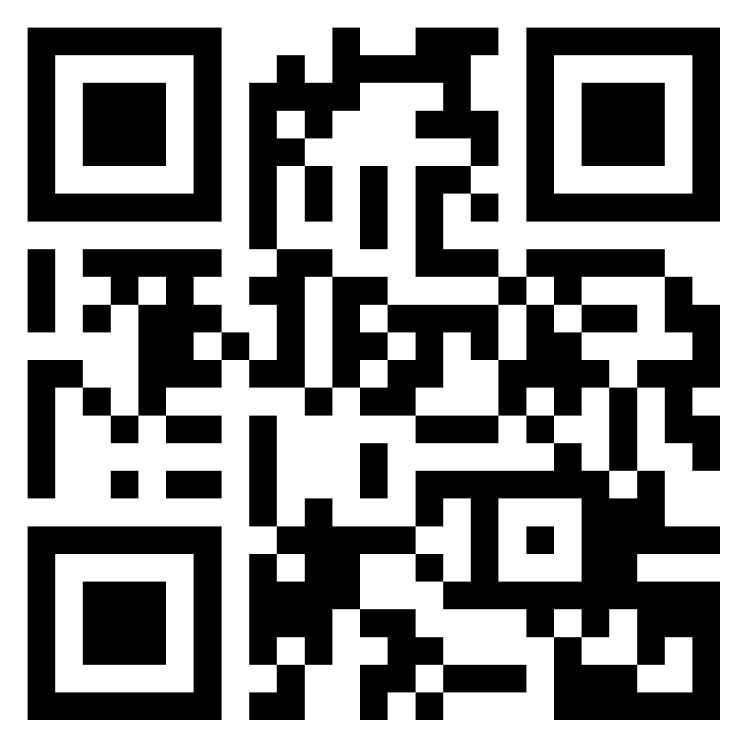

Point your SmartPhone at the code above. If you have a QR code reader the video abstract will appear. Or use:

https://youtu.be/4mLW1DbeYVw

## Introduction

Falls are a major public health issue among older people.^1^ They are of even more concern among people with dementia (PWD), who are more than twice as likely to fall and twice as likely to experience injurious falls as their cognitively intact peers.^2^,^3^ PWD admitted to hospital with a fall injury are more likely to experience adverse health outcomes during their stay and after discharge such as hospital readmission, institutionalisation, and mortality.^4^,^5^

There is robust evidence for interventions, and in particular exercise-based interventions, to prevent falls and fall-related injuries among community-dwelling people without cognitive impairment.^6^–^8^ However, to date, only three exercise trials have been conducted with community-dwelling PWD,^9^–^11^ of which only one reported outcomes up to a 12-month follow-up.^9^ This latter study used an intensive provision that may be too expensive for some health services, including the UK. Thus, there is a need for more evidence-based fall prevention interventions for PWD.

Tai Chi is an ancient form of Chinese mind–body exercise, where participants carry out smooth and continuous body movements along with deep breathing and mental concentration;^12^ equivalent to moderate-intensity exercise and quiet meditation.^13^ This form of exercise is particularly suited for PWD with its use of slow and repetitive movements.^14^ Tai Chi has been found to provide numerous health benefits,^15^ though most of the relevant research to date has focused on balance outcomes among healthy older people.^16^

We conducted a trial to test the effect of Tai Chi on improving postural balance among PWD. It was also a feasibility study for a subsequent definitive trial to test the effect of Tai Chi on preventing falls among PWD. Systematic reviews have shown that Tai Chi is an effective exercise-based intervention for preventing falls among older people,^8^ frail and at-risk older adults,^17^,^18^ and older people with Parkinson’s disease and stroke.^19^ We report the first randomised controlled trial to test if Tai Chi can improve postural balance among PWD, and the future definitive trial will be the first to test if Tai Chi can prevent falls among PWD.

## Materials And Methods

### Design

**Figure 1:**
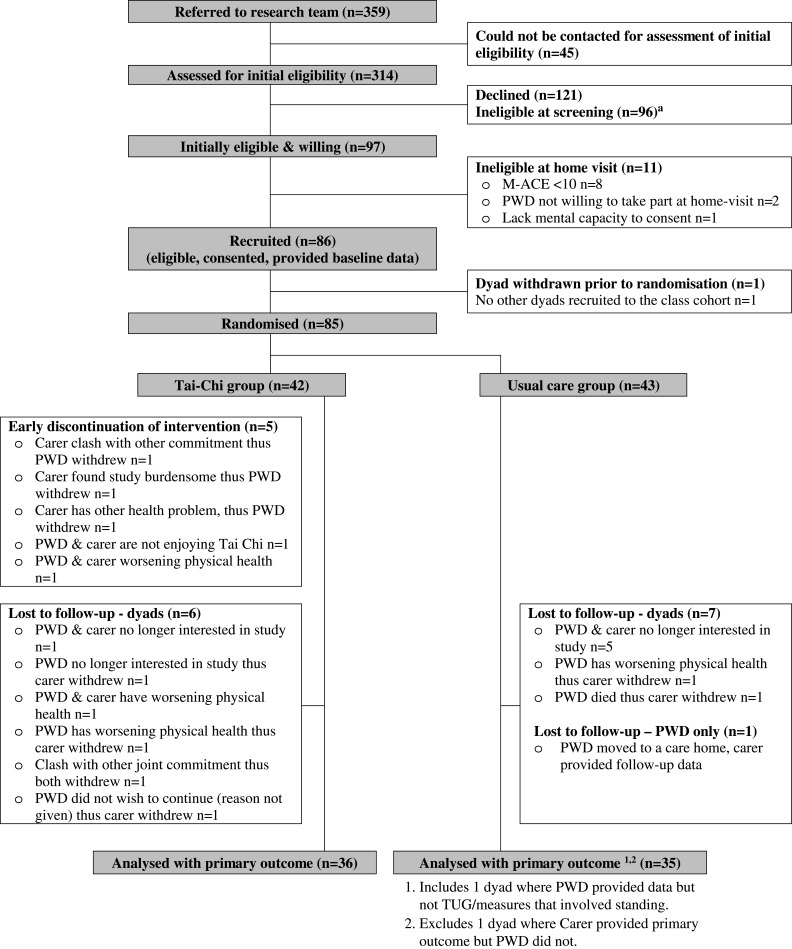
Flow diagram of study participation. **Note:** See Supplementary Figure S1 for details.

We conducted a randomised, assessor-blind, two-arm, parallel group, superiority trial. The trial is registered (ClinicalTrials.gov ID no: NCT02864056, first posted August 11^th^, 2016), and was preceded by a pilot intervention phase.^20^ The trial was approved by the West of Scotland Research Ethics Committee 4 (reference: 16/WS/0139) and the Health Research Authority (IRAS project ID: 209193). A summary of the protocol is available along with details to access the full protocol and dataset.^21^ We randomised dyads, comprising a PWD and their informal carer, to either a control group (usual care) or an intervention group (usual care plus the TACIT Tai Chi intervention) in a 1:1 ratio at three recruitment sites in the south of England (see Figure 1). Randomisation was stratified by site, and we used minimisation within each site by treatment condition and 12-month fall history at baseline (fallen/not fallen). Randomisation was processed via a centralised web-based randomisation system designed and maintained by the UKCRC-registered Peninsula Clinical Trials Unit. After completion of the baseline home visit, a member of the trials unit randomised dyads and sent them a letter to advise their treatment allocation. During the trial, to aid recruitment, we made the following protocol amendments: reduced the eligibility criteria to a minimum age of 18 years and minimum Mini Addenbrooke’s Cognitive Examination (M-ACE) score of 10, and reimbursed participants for their travel (intervention group) and participation (control group).

### Participants

Participants were identified and recruited via various sources, including National Health Service research/clinic databases, memory assessment services, local charities, and self-referral. Both the person with dementia and their informal carer were required to consent to participate. After referral, a member of the research team checked eligibility and then arranged a home visit to the dyad. At the visit, they took informed consent and then administered the M-ACE to confirm eligibility.^22^ PWD were included if they met the following criteria: aged 18 or above, living at home, had a diagnosis of dementia (indicated on their medical record held by the National Health Service or general practitioner), physically able to do standing Tai Chi, and willing to attend weekly Tai Chi classes. PWD were excluded if they met any of the following criteria: living in a care home, in receipt of palliative care, had severe dementia (baseline M-ACE score of ≤9),^22^ had a Lewy body dementia or dementia with Parkinson’s disease, had severe sensory impairment, were currently practising or had been practising within the past six months Tai Chi or similar exercise (Qi Gong, yoga, or Pilates) on average once a week or more, were currently under the care of or had been referred to a falls clinic for assessment, currently attending a balance exercise programme (eg Otago classes), or lacked mental capacity to provide informed consent. Informal carers were included if they met the following criteria: living with the PWD or could visit at least twice per week, were able to support the PWD by participating in data collection throughout the trial and in the intervention components (if randomised), able to do standing Tai Chi, and willing to attend weekly Tai Chi classes. Carers were excluded if they met any of the following criteria: had severe sensory impairment or lacked mental capacity to provide informed consent.

### Intervention

Both groups received usual care. This may have included prescribed medicine and signposting to services for information and opportunities to socialise and receive peer support, but no exercise prescription. The intervention group also received a Tai Chi intervention comprising 3 components: (1) Tai Chi classes, (2) home-based Tai Chi practice, and (3) behaviour change techniques (see Table S1). The intervention was designed for participants to accrue 50 hrs or more Tai Chi in line with evidence that higher doses of exercise lead to greater reductions in falls.^7^ Classes were held once a week in suitable venues (eg, church halls). Each session was booked for 90 mins, with 45 mins instructor-led group Tai Chi followed by up to 45 mins informal discussion. Dyads were encouraged to participate in the informal discussions each week to foster mutual peer support, and provide opportunity for ongoing advice from the Tai Chi instructor in relation to the home-based practice. Up to 10 dyads were recruited per class. The approach to teaching at each class was the repetition of movements and positive reinforcement. This approach capitalises on PWD’s capacity to continue to learn and remember motor tasks with the use of procedural or kinaesthetic memory, ie, through making behaviours automatic, despite impaired ability to explicitly recollect such memories.^23^

The 20-week course was delivered by either a lead instructor with experience in teaching PWD or an additional instructor. Both instructors were experienced in teaching Tai Chi and had qualifications at senior instructor level for public Tai Chi classes. The lead instructor observed the other instructor teach a class for one of their first cohorts to ensure fidelity and provided minor adjustment to their teaching style. Five percent of classes were observed by a researcher who completed a fidelity checklist.

### Outcomes

After demographic data were collected at baseline, the majority of measures were taken at baseline and repeated at six months post-baseline in dyads’ homes by a researcher kept blind to treatment condition. Dyads were reminded prior to the home visit to conceal their treatment allocation. Full details of the outcome measures used have been reported previously.^21^

#### Primary Outcome

For dynamic balance, we measured PWD’s mean timed up and go (TUG) score.^24^ This is a measure of how many seconds it takes for a participant to transition from a seated position to stand, walk 3 metres, turn, walk back, and be seated again.

#### Secondary Outcomes: PWD

For functional balance, we measured Berg balance score.^25^ For static balance, we measured postural sway while standing on the floor and on a foam mat,^26^ using total (anteroposterior + medio-lateral) normalised path length of the acceleration sway trace of the pelvis. This was recorded digitally using a Balance Sensor (THETAmetrix), mounted over the upper sacrum.

In a structured interview, PWD completed the Iconographical Falls Efficacy Scale (Icon-FES, short form)^27^ and the ICEpop CAPability measure for Older people (ICECAP-O)^28^ for fear of falls and quality of life, respectively. As noted above, they also completed the M-ACE as a measure of global cognitive functioning.^22^

Falls among PWD were collected prospectively from baseline until the follow-up home visit.^29^ We defined a fall as, ‘‘an unexpected event in which the participants come to rest on the ground, floor or lower level”.^29^ Falls were recorded prospectively by dyads daily, using calendars returned on a monthly basis by post. Telephone calls by an unblinded research assistant were conducted weekly to collect falls data as well,^30^ along with further information about falls and adverse events from dyads in the intervention group. To ascertain the accuracy of different recall periods, the research assistant conducted telephone calls about fall incidents by the PWD (monthly with the PWD and every 3 months with the carer). Each method of data collection was amalgamated into one overall measure of fall incidence, with duplicates removed (based on dates and description of the fall events).^30^ Fall injury was recorded by telephone interview when recording falls using existing definitions,^31^ as was health service use in relation to falls or adverse events. The total cost of providing the intervention to each patient was estimated from weekly registers completed by the Tai Chi instructors.

#### Secondary Outcomes: Informal Carers

Carers supported PWD in the study with data collection, and in the intervention arm, with their home practice of Tai Chi. To enable carers to facilitate Tai Chi home practise, they attended and participated in the Tai Chi classes along with the PWD. Therefore, we hypothesised that carers would also benefit from the Tai Chi intervention and tested for this. Carers completed the TUG and postural sway tests as described above. They also self-completed, away from the PWD, the ICECAP-O and Zarit Burden Interview (short-form).^32^

### Statistical Analysis

#### Sample Size

The sample size was based on an estimated smallest detectable change on the TUG of a value of 4,^33^,^34^ standard deviation of 9.38,^34^ and correlation with baseline score of 0.7. Using the above values and a 2-sided 5% significance level, the study would have 90% power with a sample size of 120. Allowing for up to 20% withdrawal/non-completion of outcome measures, we aimed to recruit 150 dyads into the trial (75 per group).

#### Analysis

Participants were analysed in the group they were randomised to on an intention-to-treat basis. The primary and secondary outcomes were compared between the two trial arms using a mixed (multi-level) model approach to take into account clustering within Tai Chi classes, baseline scores, treatment site, and 12-month falls history. Fall incidence and the proportion of participants who fell were analysed similarly using negative binomial and logistic models, respectively. In addition, we conducted a per protocol analysis that excluded two people who did not have a dementia diagnosis (protocol violations) and participants from the Tai Chi group if they received fewer than 34 hrs. We also conducted a pre-planned subgroup analysis on mean TUG scores at 6-month follow-up according to baseline fall history.

## Results

### Participants

Dyads were recruited from 06/04/2017 to 17/07/2018, with the final follow-up completed on 30/11/2018. Figure 1 displays the recruitment and retention of participants (see Figure S1 for reasons declined/ineligible). Of the 359 approached, 85 dyads participated (24%), of which 70 (82%) had complete data for the primary outcome variable. Baseline characteristics suggested an even balance across trial arms including medication consumption and other long-term health conditions (see Table 1, and Tables S2–3 for further details).

**Table 1:** Baseline Descriptive Statistics

	Usual Care Group (n=43)	Tai Chi Group (n=42)
People with dementia		
Female, n (%)	16 (37%)	18 (43%)
Age mean (SD), range	78.2 (7.5) 61.9–97.4	77.9 (8.3) 59.0–88.0
Type of dementia, n (%)		
Alzheimer’s	26 (60%)	30 (71%)
Vascular	5 (12%)	1 (2%)
Alzheimer’s and vascular	6 (14%)	9 (21%)
Other	6 (14%)	2 (5%)
Time since diagnosis (years) median (IQR)	1.4 (2.6) 0.1–7.5	1.1 (2.5) 0.2–7.7
Fallen in past 12 months, n (%)	18 (42%)	19 (45%)
Recruitment site, n (%)		
National Health Service 1	11 (26%)	10 (24%)
National Health Service 2	30 (70%)	30 (71%)
National Health Service 3	2 (5%)	2 (5%)
Informal carers		
Female, n (%)	35 (81%)	32 (76%)
Age mean (SD) range	70.8 (10.4) 47.5–88.8	72.0 (9.9) 43.4–87.9
Living with PWD, n (%)	38 (88%)	36 (86%)

### Fidelity Of Intervention Delivery

Thirty-four classes were observed and almost all aspects of the intervention were consistently delivered. The exceptions were that refreshments were not always provided to encourage socialising after classes, particularly when classes finished late in the afternoon or where parking was restricted. While the instructors emphasised the importance of Tai Chi home practice, they did not emphasise the intended dose of 20 mins per day.

### Adherence

Out of a total possible 678 class attendances, there were 457 attendances by PWD and 449 by carers. Mean attendance was 11 classes for both PWD (SD = 6.46, n=41) and carers (SD = 6.68, n =41), or 8.4 and 8.2 hrs, respectively. Mean adherence to home practice was 35% (SD = 30.5, n=38), or 16.5 hrs (SD = 15.14, n=38) for PWD and 17 hrs (SD = 16.55, n=38) for carers. Mean dose of Tai Chi was 23.6 hrs (SD = 19.27, n=41) for PWD and 24.1 hrs (SD = 20.84, n=41) for carers. Three participants (7%) received the intended 50 hrs dose.

### Outcomes At Follow-Up: PWD

The outcomes for PWD at follow-up are shown in Tables 2 and 3. There was no significant between-group difference on the TUG in the primary analysis or pre-planned subgroup analysis between those with/without a falls history at baseline. Among the secondary outcomes, PWD in the Tai Chi group had a significantly higher quality of life (medium effect size) and a significantly lower rate of falls (medium effect size, though sensitive to an outlier). The remaining secondary outcomes were not significant with little difference between trial arms. Per protocol analysis obtained similar results.

**Table 2:** Continuous Outcomes For People With Dementia And Their Informal Carers

	Baseline	6-Month Follow-Up	Mean Difference (95% CI) At Follow-Up
People with dementia			
Primary outcome: Timed up and go test mean (SD)^a^			
Usual care group	18.7 (6.4), n=43	19.7 (5.3), n=34	0.82 (−2.17, 3.81) *p* = 0.59, *d* = 0.14
Tai Chi group	18.5 (5.1), n=42	21.1 (8.7), n=36
Secondary outcomes mean (SD)			
Berg Balance Scale^b^			
Usual care group	44.5 (6.8), n=43	44.7 (7.2), n=32	−0.01 (−1.86, 1.83) *p* = 0.99, *d* = −0.002
Tai Chi group	45.9 (5.4), n=42	44.8 (5.7), n=36
Postural sway standing on floor (mg/s)^c^			
Usual care group	166 (43), n=43	164 (22), n=34	1.0 (−14.09, 16.10) *p* = 0.90, *d* = 0.03
Tai Chi group	157 (23), n=42	161 (38), n=36
Postural sway standing on foam (mg/s)^c^			
Usual care group	210 (75), n=43	205 (62), n=34	−6.17 (−29.15,16.82) *p* = 0.60, *d* = −0.09
Tai Chi group	209 (63), n=42	198 (46), n=36
Iconographical Falls Efficacy Scale^d^			
Usual care group	16.1 (6.1), n=43	18.2 (7.2), n=34	−1.53 (−4.43, 1.38) *p* = 0.30, *d* = −0.25
Tai Chi group	16.6 (6.0), n=42	17.3 (6.3), n=36
ICEpop CAPability measure for Older people^e^			
Usual care group	0.88 (0.11), n=43	0.83 (0.14), n=34	0.051 (0.002, 0.100) *p* = 0.04, *d* = 0.51
Tai Chi group	0.87 (0.09), n=42	0.86 (0.10), n=36
Mini-Addenbrooke’s Cognitive Examination^f^			
Usual care group	15.1 (4.3), n=43	13.7 (6.3), n=35	−0.35 (−2.20, 1.49) *p* = 0.71, *d* = −0.08
Tai Chi group	16.2 (4.9), n=42	14.5 (6.4), n=36
Informal carers			
Secondary outcomes mean (SD)			
Timed up and go test^a^			
Usual care group	13.6 (3.5), n=43	13.9 (2.8), n=36	1.83 (0.12, 3.53) *p* = 0.04, *d* = 0.61
Tai Chi group	13.0 (2.4), n=42	15.5 (5.9), n=36
Postural sway standing on floor (mg/s)^c^			
Usual care group	150 (15), n=43	154 (14), n=36	−4.11 (−10.13, 1.90) *p* = 0.18, *d* = −0.32
Tai Chi group	152 (11), n=42	150 (12), n=36
Postural sway standing on foam (mg/s)^c^			
Usual care group	173 (26), n=43	166 (20), n=36	2.16 (−10.96, 15.28) *p* = 0.75, *d* = 0.09
Tai Chi group	170 (20), n=42	168 (32), n=35
ICEpop CAPability measure for Older people^e^			
Usual care group	0.86 (0.11), n=43	0.79 (0.12), n=34	−0.003 (−0.050, 0.044) *p* = 0.90, *d* = −0.03
Tai Chi group	0.83 (0.11), n=41	0.78 (0.13), n=35
Zarit Burden interview (short-form)^g^			
Usual care group	15.5 (7.4), n=43	17.7 (8.4), n=35	0.52 (−1.93, 2.96) *p* = 0.68, *d* = 0.06
Tai Chi group	16.9 (9.8), n=41	18.8 (9.4), n=35

**Notes:** ^a^Lower values indicate greater dynamic balance. Mean [SD] seat height at baseline was to standard for the test (46 cms/arm rest height 67 cms, n=43) for usual care (46.6 [3.4]/65.6 [5.0], for n=25 with arm rest) and Tai Chi groups (45.7 [2.7]/65.3 [2.5], for n=18). ^b^Higher scores indicate greater functional balance, potential range 0–56. ^c^Higher scores indicate worse static balance. ^d^Higher scores indicate greater concern, potential range 10–40. ^e^Higher scores indicate better capability. ^f^Higher scores indicate greater cognitive functioning, potential range 0–30. ^g^Higher scores indicate greater burden, potential range 0–48.

**Table 3:** Falls Outcomes For People With Dementia

	Usual Care Group	Tai Chi Group	Ratio At Follow-Up (95% CI)
Number of falls (number per month of follow-up)^a^			
6-month follow-up^b^	78 (0.312), n=43	44 (0.174), n=42	Falls rate ratio: 0.35 (0.15, 0.81) *p* = 0.015
Number of injurious falls (number per month of follow-up)^a^			
6-month follow-up	17 (0.068), n=43	11 (0.043), n=42	Falls rate ratio: 0.62 (0.23, 1.66) *p* = 0.34
Proportion of participants falling^c^			
6-month follow-up	17 (47%), n=36	17 (47%), n=36	Odds ratio: 0.97 (0.28, 3.33) *p* = 0.96
Proportion of participants having an injurious fall^c^			
6-month follow-up	8 (22%), n=36	9 (25%), n=36	Odds ratio: 1.09 (0.33, 3.56) *p* = 0.89

**Notes:** ^a^Follow-up (min, max), median months = (0.30, 8.25), 6.41. Calculation of falls rate takes into account length of follow-up and so includes all participants. ^b^One person with dementia in the control group had 17 falls. When this participant was excluded from the analysis, the falls rate ratio changed to 0.46 (95% CI = 0.21, 1.03), *p* = 0.060. Hypothetically, if this one person had been randomised to the Tai Chi group instead of the control group and they had not participated in the intervention, and they again had 17 falls, then the intention to treat analysis would suggest that the number of falls in each group would have been identical. However, in this hypothetical scenario, the per protocol analysis would exclude this individual and so the incidence of falls would then be as above with a falls rate ratio of 0.46 (95% CI = 0.21, 1.03), *p* = 0.060. ^c^Calculation of proportion of fallers only includes those who were followed up at 6 months.

### Outcomes At Follow-Up: Informal Carers

The outcomes for carers at follow-up are shown in Table 2. Carers in the Tai Chi group had significantly worse performance on the TUG (medium effect size). The remaining secondary outcomes were not significant with little difference between trial arms. Per protocol analysis obtained similar results.

### Adverse Events

No serious adverse events were related to participation in the trial (see Table S4).

### Health Economics

The cost of Tai Chi instructors came to £26,995, with a mean cost of £631 per intervention group dyad. This was markedly higher than dyads’ willingness to pay (see Table S5).

### Assessor Blinding At Follow-Up

The outcome assessor was accidentally unblinded at follow-up by 9 dyads. The assessor was then able to correctly guess their treatment allocation, and guess correctly 63% of treatment allocations (45/72, p=0.044).

## Discussion

This randomised controlled trial showed that compared to usual care alone, Tai Chi in addition to usual care did not improve postural balance among PWD. This was evident from both the primary outcome (TUG) and secondary outcomes (Berg balance and postural sway). PWD in the Tai Chi group had a significantly greater quality of life (standardised effect size = 0.51). There was a trend for a reduction in falls among PWD in the Tai Chi group, which became non-significant (*p* = 0.06) once an outlier was removed. There were no significant improvements for PWD on the other secondary outcomes. For carers, the Tai Chi group had significantly worse TUG scores (standardised effect size =0.61) but no significant change in postural sway. Carrying out and supporting PWD to participate in Tai Chi led to no significant change in their quality of life or carer burden. The above marginal statistically significant secondary outcomes need to be interpreted in the context of 15 secondary outcomes and the risk of type 1 error. While the power for the statistical analysis of the primary outcome was lower than planned due to under-recruitment, the 95% confidence interval did not include the smallest detectable change of 4 and therefore any real difference between groups at follow-up on the TUG is unlikely to be of clinical importance. Tai Chi was found to be safe with no serious adverse events experienced in relation to practising Tai Chi in class or at home.

### Primary And Secondary Outcomes: PWD

Our results contrast with previous studies that have found Tai Chi to improve scores on the TUG among older people (weighted mean difference [WMD] = 1.04, 95% CI: 0.67, 1.41)^35^ and people with Parkinson’s disease when compared to a no-treatment group (WMD = −2.13, 95% CI: −3.26, −1.00).^19^ In addition, our results contrast with previous findings for Tai Chi to improve Berg balance scores among older people (WMD = 2.86, 95% CI: 1.91, 3.81),^35^ and improve static balance among those at low but not high risk of falling.^36^ However, these previous improvements may not be clinically significant,^37^,^38^ suggesting that Tai Chi may prevent falls through other mechanisms and not primarily through static and dynamic balance. Given that Tai Chi promotes slow and mindful movement, it may be that the intervention group were walking more mindfully and so at less risk of falls. Further research could examine whether Tai Chi leads to clinically and statistically significant improvements on other outcomes not measured such as leg muscle strength.

We hypothesised that the mechanism for Tai Chi to reduce falls would be via an improvement in postural stability. While we did not observe a significant reduction in the number of fallers, this was less likely as previous exercise interventions have reduced the rate of falls by an average of 23% but the number of fallers by 15%.^8^ Similarly, we did not observe a significant reduction in injurious falls, as they have a lower event rate and would need a large sample to identify a treatment effect.^31^ However, we identified a trend for a reduction in the rate of falls among the Tai Chi group. This trend was no longer significant when an outlier with a high rate of falls in the control group was removed (see footnote, Table 3). Future trials of Tai Chi and other exercise-based interventions should examine the mechanism(s) for a reduction in falls. This would build on a trial that found Tai Chi reduced falls more effectively than multi-modal exercise, but no secondary outcomes were different between the two arms to explain the mechanism.^39^ It would also build on a previous exercise trial that found a reduction in falls without an improvement in the TUG and functional reach tests.^40^ Other possible mechanisms would include improving leg muscle strength and cognitive motor control to perform everyday activities safely such as stepping onto a curb,^41^,^42^ and improving cognition to be more able to complete two tasks at the same time, such as walking while talking.^43^,^44^

We found no significant improvement for PWD in the Tai Chi group on fear of falls or global cognitive functioning. While there is weak evidence that exercise reduces fear of falls post-intervention,^45^ our findings contrast with previous studies that have found Tai Chi to enhance cognitive functioning among those with and without dementia.^46^ Further research could examine the benefits of Tai Chi using more sensitive and specific measures of cognitive functioning such as executive functioning.

We found quality of life to be significantly higher among PWD in the Tai Chi group. Previous studies have found that Tai Chi improves physical and mental health-related quality of life,^12^ including depression, anxiety, and psychological well-being.^47^ However, our results suggest that the Tai Chi group retained their level of quality of life and the control group significantly worsened. It is possible that the worsening in quality of life observed in the control group was associated with their trend for a greater rate of falls. Alternatively, PWD may have retained their quality of life through the benefits of Tai Chi from its use of mindfulness, relaxation, cognitive stimulation, and social interaction.^48^

While the reporting of adverse events in previous Tai Chi trials has been poor and inconsistent, our study supports the evidence base that Tai Chi does not lead to serious adverse events (eg, a fall resulting in hip fracture) but may be associated with some minor and expected adverse events (eg, knee and backache).^49^

### Secondary Outcomes: Informal Carers

It is unclear why we found carers in the Tai Chi group to have significantly worse TUG scores. Due to unblinding of the assessor early in the trial, we removed questions from the exit interview on exercise conducted outside of the provided intervention. It could be that carers in the control group engaged in more exercise that improved their balance due to disappointment of not being randomised to Tai Chi. Future research should measure physical activity in conjunction with measures of balance and falls to clarify causal effects.^50^ Alternatively, the intervention may have increased carers’ awareness of the risk of falls and to walk “more mindfully”, and so they may have walked slower but more safely. Future research would benefit from using other measures of physical functioning that do not rely on gait speed.

We found no evidence for change in quality of life or carer burden among carers. This contrasts with previous studies that found improvements in carer burden and quality of life among carers supporting PWD participating in an exercise or cognition-based intervention, respectively;^51^,^52^ but greater anxiety and stress among carers supporting PWD with reminiscence therapy.^53^ Perhaps the lack of change on these variables observed in this study was because the additional demands on carers to facilitate Tai Chi class attendance and home practice were balanced by the enjoyment of these activities. Future research could qualitatively explore this in more detail.

### Study Limitations

While this was a pragmatic trial and the eligibility criteria were kept as broad as possible, the effect of Tai Chi found in our study may be weaker when applied to the general population of PWD and their informal carers. This trial was limited by a reduction in statistical power due to a lower number of dyads recruited than expected. This is reflective of the broader challenges of recruiting and retaining PWD and their informal carers in research and the need to recruit dyads in groups within the trial design. The reduction in statistical power for detecting differences in all the outcomes, including the TUG from 90% to 69%, means that it is possible the study missed important effects (eg, rate of falls once the outlier was removed). However, we note that the smallest detectable change of a value of 4 s for the TUG was outside the 95% confidence interval (−2.17, 3.81), suggesting our test on the primary outcome was adequately powered.

The study was also limited by the Tai Chi group receiving a lower dose than planned. However, the exact dose needed to prevent falls is unknown. Indeed, current knowledge on intervention dose is drawn from a meta-regression across various interventions and contexts and not specifically, eg, Tai Chi for PWD.^7^ Class attendance and home practice was comparable to prior exercise trials, though slightly lower in this study given the previous studies excluded PWD.^54^–^56^ Further research is required to determine the exact dose required of specific exercise interventions to prevent falls in specific populations. Another limitation is that we did not collect data to confirm the homework sheets were used for the Tai Chi home practice. Future research could collect data to confirm not only the quantity of home practice but also the quality (eg, which exercises were performed each week).

### Practice Implications

While practitioners await evidence from future robust definitive trials as to the clinical and cost-effectiveness of Tai Chi for preventing falls among PWD, this study demonstrates that Tai Chi is a safe activity for PWD. This study also suggests that the support required from carers does not decrease their perceived quality of life or increase their perceived carer burden. Indeed, our earlier work found the intervention to be acceptable to PWD and their carers.^20^ Therefore, qualified Tai Chi instructors are encouraged to provide classes for PWD and their family carers so that PWD may also benefit from this exercise for their general health and wellbeing.^57^,^58^

## Conclusions

The results suggest that there is potential for Tai Chi as a safe exercise intervention to reduce falls among community-dwelling PWD and improve their quality of life. Also, the intervention did not increase carer burden or reduce quality of life among informal carers. Further work is required to increase adherence to the home-based element of the intervention and identify the mechanism(s) for its potential to reduce falls.

## Data Availability

The electronic, quantitative trial data will be shared with bona fide researchers intending to use the data for non-commercial research purposes, after an embargo period of approximately 24 months (ending January 2021). Access to the following will be restricted to researchers who sign a confidentiality agreement and confirm their intention to use the data is for secondary data analysis for non-commercial research purposes using a Creative Commons licence: statistical analysis plan; where applicable, statistical code (for final analysis of primary outcome measure); and anonymised participant-level dataset and data documentation. Interested parties may make a formal request to access the electronic dataset, which will be approved/declined by the chief investigator in accordance with the Data Management Plan that will detail management of access, sharing, and preservation of the data. Any use of the electronic data set must be requested via Bournemouth University Library (bordar@bournemouth.ac.uk) who will collaborate with the chief investigator with regards to access.

## Abbreviations

CI, Confidence Interval; ES, Effect Size; ICECAP-O, ICEpop CAPability measure for Older people; Icon-FES, Iconographical Falls Efficacy Scale; M-ACE, Mini Addenbrooke’s Cognitive Examination; MD, Mean Difference; PWD, People With Dementia; SD, Standard Deviation; TUG, Timed up and Go test; WMD, Weighted Mean Difference.
